# The influence of canine parvovirus infection on the metabolome of captive Chinese and Malaysia pangolins

**DOI:** 10.3389/fcimb.2026.1910730

**Published:** 2026-09-08

**Authors:** Chengwei Xiang, Jingling Yu, Yiran Hu, Fanghui Hou, Yinhua Li, Luman Chen, Mingzheng Han

**Affiliations:** 1 Guangdong Provincial Wildlife Monitoring and Rescue Center, Guangzhou, China; 2 College of Veterinary Medicine, South China Agricultural University, Guangzhou, China

**Keywords:** canine parvovirus, fecal metabolomics, interspecific difference, pangolin, untargeted metabolomics

## Abstract

Canine parvovirus (CPV) infection poses a serious threat to pangolins, but the characteristics of the host response at the metabolic level remain unclear. In this study, we used non-targeted fecal metabolomics to systematically analyze differences in fecal metabolites in the Malaysian pangolin and the Chinese pangolin before and after CPV infection. The results indicated that CPV infection significantly altered the fecal metabolic profiles of both pangolin species. In the Malaysian pangolin, post-infection differential metabolites were primarily enriched in lipid metabolic pathways, such as α-linolenic acid and arachidonic acid metabolism, as well as inflammation-related pathways, including ferroptosis and necrotic apoptosis. Whereas the Chinese pangolin exhibited not only lipid metabolism dysregulation but also amino acid metabolic reprogramming and bile acid metabolism dysregulation. There were significant differences in metabolic responses between the two species, with the Malaysian pangolin characterized by lipid metabolism abnormalities, while the Chinese pangolin showed a greater focus on amino acid and bile acid metabolism. This study revealed the specific metabolic response of pangolins infected with CPV, provided potential fecal metabolic biomarkers for the early identification of CPV infection in pangolins, and provided a new theoretical basis for disease monitoring and intervention in pangolin conservation medicine.

## 1 Introduction

Pangolins belong to the order *Pholidota* and are the only extant group of mammals whose entire bodies are covered with keratinous scales. There are currently eight extant species of pangolins worldwide, distributed across Asia and Africa. In Asia, these include four species: the Chinese pangolin, the Malaysian pangolin, the Indian pangolin, and the Philippine pangolin. Pangolins are insectivorous animals that play an indispensable ecological role in maintaining the balance of termite and ant populations within forest ecosystems (Gu et al., 2024). However, due to illegal trade, habitat loss, and low reproductive rates, all eight pangolin species have been listed under Appendix I of the Convention on International Trade in Endangered Species of Wild Fauna and Flora (CITES), prohibiting international commercial trade in related derivatives. The IUCN Red List classifies the Chinese pangolin and the Malaysian pangolin as “Critically Endangered”. Research indicates that nearly 91% of areas suitable for Malaysian pangolins in Sabah are vulnerable to poachers, presenting a severe conservation challenge (Panjang et al., 2024). In the 1960s, the population of Chinese pangolins was estimated at around 800,000, this number had declined to approximately 16,000 by 2020 (Hu et al., 2026). Consequently, *ex situ* conservation and captive rescue have become critical strategies for pangolin conservation. Nevertheless, in captivity, pangolins face multiple health threats including malnutrition, stress, and infection by pathogenic microorganisms (Dai et al., 2025; Hua et al., 2015), among which infectious diseases deserve particular attention.

Canine parvovirus (CPV) is an unenveloped single-stranded DNA virus belonging to the *Parvoviridae*. The virus primarily infects canids, causing severe hemorrhagic enteritis, leukopenia, and even death by attacking rapidly dividing intestinal epithelial cells and lymphocytes (Li et al., 2025). Recent studies indicate that the host range of CPV is continuously expanding. Research conducted in the Newfoundland and Labrador region of Canada found that CPV-2 was detected in free-roaming dogs (2/48, 4.2%) and wild coyotes (3/87, 3.5%), and the strains were highly homologous, indicating that the virus can cross species and spread between wild and domestic carnivores (Canuti et al., 2022). Furthermore, the CPV-2c subtype has been confirmed to infect a variety of wild animals (Sarchese et al., 2025), thereby further expanding the host range of this virus. CPV is highly environmentally stable and extremely contagious. It spreads through the fecal-oral route and is highly likely to cause outbreaks in wildlife rescue, sheltering or breeding environment. In recent years, cases of pangolin infection with CPV have been confirmed at the pathogen level. Zhang’s team (Lina et al., 2022) detected CPV-2a and CPV-2c in two rescued *Manis pentadactyla* in southern China. Both pangolins exhibited severe diarrhea symptoms and ultimately died. Pathological examination showed that, similar to dogs infected with CPV-2, a prominent feature of pangolin infection was severe lesions in the small intestinal mucosa. Immunohistochemistry confirmed the distribution of CPV-2 antigens in the small intestinal crypts. Phylogenetic analysis indicated that the CPV-2c strain isolated from Taiwanese pangolins had 99.8% sequence identity with the strain isolated from Chinese mainland pangolins, suggesting that the virus may be spreading among the pangolin populations. Additionally, clinical records from domestic rescue institutions also showed that cases of death due to small virus infection occurred among the rescued pangolins, presenting typical symptoms such as hemorrhagic enteritis (Lin et al., 2026).

Although relevant studies have confirmed that pangolins can be infected with CPV, there is still a lack of systematic research on the metabolic response characteristics of pangolins after viral infection. Viral infection can cause intestinal microecological disorders and metabolic reprogramming in the host (Wu et al., 2026), and fecal metabolomics, as a non-invasive and sensitive analytical method, can effectively reflect functional changes during host-pathogen interactions. By analyzing differences in fecal metabolites, it is possible to reveal the effects of viral infection on host energy metabolism, amino acid metabolism, lipid metabolism and other pathways, providing important clues for the early identification of diseases and the interpretation of pathological mechanisms. Therefore, this study focuses on the Malaysian pangolin and the Chinese pangolin. Using non-targeted fecal metabolomics technology, we compare and analyze differences in fecal metabolites between CPV-positive individuals and healthy controls, as well as between the two pangolin species following CPV infection. CPV infection significantly changed the fecal metabolic profiles of the two pangolins, and the differential metabolites were mainly enriched in lipid metabolism, amino acid metabolism, and inflammation-related pathways. Malaysian pangolins were characterized by lipid metabolism disorders, while the Chinese pangolins were more prominent in amino acid and bile acid metabolism disorders, suggesting different metabolic reprogramming patterns in the two species after CPV infection. The differential metabolites were screened and related metabolic pathways were enriched, aiming to reveal the metabolic response characteristics of CPV infection in pangolins and provide theoretical basis for the early diagnosis of pangolin infectious diseases.

## 2 Materials and methods

### 2.1 Animal population

The pangolin samples analyzed in this study were rescued by local government officials and members of the public in Guangdong Province and transported to the Guangdong Provincial Wildlife Rescue and Testing Center. Each animal was quarantined for at least one month, and injured or ill individuals were treated until apparent recovery. The criteria for healthy controls were active, good appetite, normal stool, absence of parasites or trauma, and stable weight. CPV-infected individuals presented with clinical symptoms such as diarrhea, vomiting, and loss of appetite, and the fecal samples were positive for CPV.

This study included 28 adult Chinese pangolins (weighing 3.1-5.96 kg) and 26 adult Malaysian pangolins (weighing 2.94-8.87 kg). Six individuals of similar body length and weight were selected from each species, with three assigned to the CPV-infected group and three to the healthy control group. Fresh fecal samples were collected from these individuals for subsequent experimental analysis.

### 2.2 Experimental reagents

The experimental reagents and instruments used in this study are listed in **Tables 1** and **2**.

**Table 1:** List of experimental reagents.

Name	CAS	Purity	Brand
Methanol	67-56-1	LC-MS	Fisher
Acetonitrile	75-05-8	LC-MS	Fisher
Isopropanol	67-63-0	LC-MS	Fisher
Acetic acid	64-19-7	LC-MS	Sigma
Ammonium acetate	631-61-8	LC-MS	Sigma
deionized water (ddH2O)	–	–	Watsons

**Table 2:** List of experimental instruments.

Instrument	Model	Brand
Ultra-high performance liquid chromatography (UPLC)	Vanquish	Thermo
High-resolution mass spectrometer (HRMS)	Q Exactive PLUS/Orbitrap Exploris 120/Orbitrap Exploris 480	Thermo
Refrigerated centrifuge	centrifuge 5427 R	Eppendorf
Vortex mixer	MS3 digital	IKA
Electronic balance	ES105A	Lichen
Low-temperature grinding mill	JXFSTPRP-CLN	Jingxin
Constant temperature ultrasonic cleaner	XM-1200UDF	Jingxin
Cryogenic concentrator	JX-ZLN-B	Jingxin

### 2.3 Metabolite extraction

Each fecal sample (20 mg) was randomly collected, placed on dry ice, and homogenized at 60 Hz for 60 seconds using liquid nitrogen. Then, 200 μL of pre-cooled H_2_O was added to a 2 mL grinding tube containing steel beads, and the mixture was ground at 60 Hz for 60 seconds. Subsequently, 800 μL of methanol/acetonitrile (1:1, v/v) was added, followed by vortexing for 30 seconds and ultrasonication at 4 °C for 10 minutes. After incubation at -20 °C for 1 hour, the sample was centrifuged at 13,000 rpm and 4 °C for 15 minutes to precipitate proteins. A 720 μL aliquot of the supernatant was transferred to a 1.5 mL centrifuge tube and concentrated to dryness under vacuum at 4 °C. The residue was reconstituted with 100 μL of acetonitrile/water (1:1, v/v), vortexed for 30 seconds, and ultrasonicated at 4 °C for 10 minutes. After centrifugation for 15 minutes, the supernatant was transferred to a sample vial with an insert and stored at 4 °C for UPLC-HRMS analysis.

### 2.4 UPLC-MS/MS

Non-targeted metabolomics analysis was performed using ultra-high performance liquid chromatography-tandem mass spectrometry (UPLC-MS/MS). Briefly, chromatographic separation was carried out on a Thermo Vanquish Flex UPLC system equipped with a Phenomenex Kinetex C18 column (2.6 μm, 2.1 × 100 mm). Mobile phase A consisted of water containing 0.01% acetic acid, and mobile phase B consisted of 50% acetonitrile and 50% isopropanol. The column temperature was set at 25 °C, the sample tray was maintained at 4 °C, and the injection volume was 2 μL. Mass spectrometric detection was performed using a Thermo Orbitrap Exploris 120 mass spectrometer. Data acquisition was conducted in full scan mode with positive and negative ion switching, while quality control (QC) samples were analyzed using full scan combined with data-dependent acquisition (DDA) mode. Key mass spectrometry parameters were as follows: sheath gas flow rate, 50 Arb; auxiliary gas flow rate, 15 Arb; capillary temperature, 350 °C; spray voltage, 3.5 kV in positive ion mode and -2.8 kV in negative ion mode.

### 2.5 Information analysis

Mass spectrometry data were preprocessed using the XCMS software for peak picking, peak grouping, retention time correction, and adduct annotation. After converting the raw data to mzXML format, the data were processed using the XCMS and metaX toolkits in R. Ions were identified based on retention time and mass-to-charge ratio (m/z), generating a three-dimensional matrix containing peak indices, sample names, and ion intensities. Metabolite annotation was performed using online KEGG and HMDB databases, as well as a custom database, with a mass deviation of less than 10 ppm as the matching criterion. Data preprocessing included filtering, KNN imputation, and PQN normalization. Clustering heatmaps were generated using the R package pheatmap, while the metaX and ropls packages were used to perform principal component analysis (PCA), partial least squares discriminant analysis (PLSDA), and variable importance projection (VIP), respectively. Significantly differentially expressed metabolites were selected based on a P-value < 0.05, fold change (FC) > 1.2, and VIP ≥ 1. Hypergeometric tests were used for KEGG pathway enrichment analysis, with a significance threshold of P < 0.05; GSEA software was used for metabolite set enrichment analysis, with significance criteria of |NES| > 1, P < 0.05, and FDR < 0.25. Network diagrams were constructed based on the pathways in which the metabolites are located.

## 3 Results

### 3.1 Analysis of metabolite differences between CPV infection in Malaysian pangolin and healthy individuals

**Figure 1:**
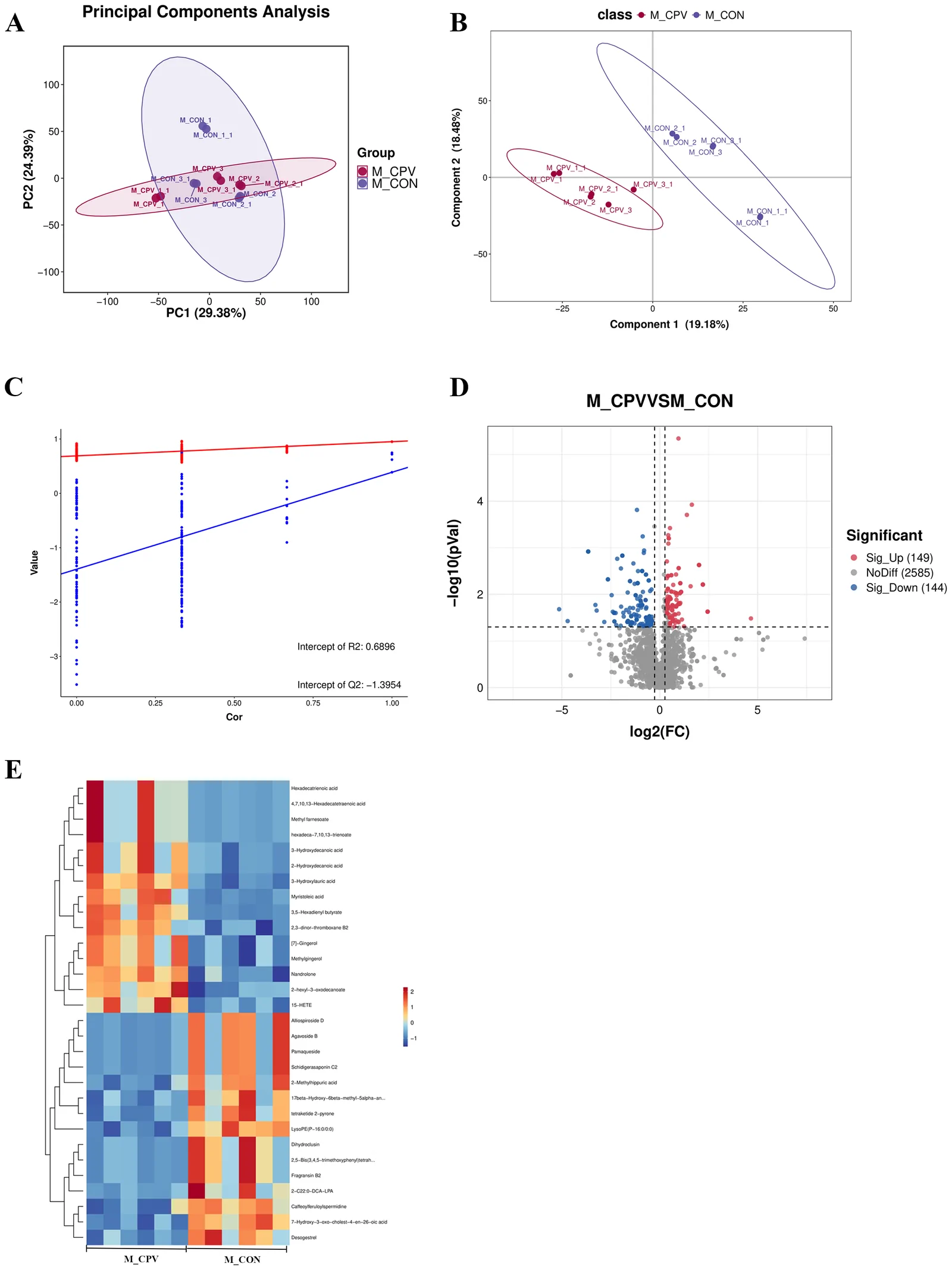
Differential analysis of metabolites between CPV-infected and healthy individuals in Malaysian pangolins. **(A)** PCA analysis. **(B, C)** PLSDA analysis and permutation test plot. **(D)** Volcano map of differential metabolites. **(E)** Heat map of differential metabolites.

Pattern discrimination analysis of different groups was performed using principal component analysis (PCA) and partial least squares discriminant analysis (PLSDA). The first principal component (PC1) explained 29.38% of the variance, and the second principal component (PC2) explained 24.39% of the variance, with the two groups showing a clear separation trend in the score plot (**Figures 1A, B**). In the PLSDA permutation test plot, the red line represents the R2 regression line, and the blue line represents the Q^2^ regression line. The x-coordinates of the permutation test results fell within the [0, 1] interval, with the R² regression line located above the Q² regression line. Additionally, the intercept of the Q² regression line with the Y-axis was less than 0 (Q² intercept = -1.3954), indicating no overfitting of the model (**Figure 1C**). The PLSDA score plot visually reflected the degree of dispersion among samples through the relative positions of the data points. The results in **Figure 1B** showed significant differences in metabolite expression patterns between the CPV-infected group (M_CPV) and the control group (M_CON). Using the screening criteria of FC > 1.2 and P < 0.05, a total of 293 significantly differential metabolites were identified, of which 149 were upregulated and 144 were downregulated in the CPV-infected group (**Figure 1D**). The heatmap of differentially expressed metabolites displayed distinct clustering characteristics between the two groups (**Figure 1E**).

### 3.2 Functional analysis of differential metabolites in Malaysian pangolins infected with CPV and healthy individuals

**Figure 2:**
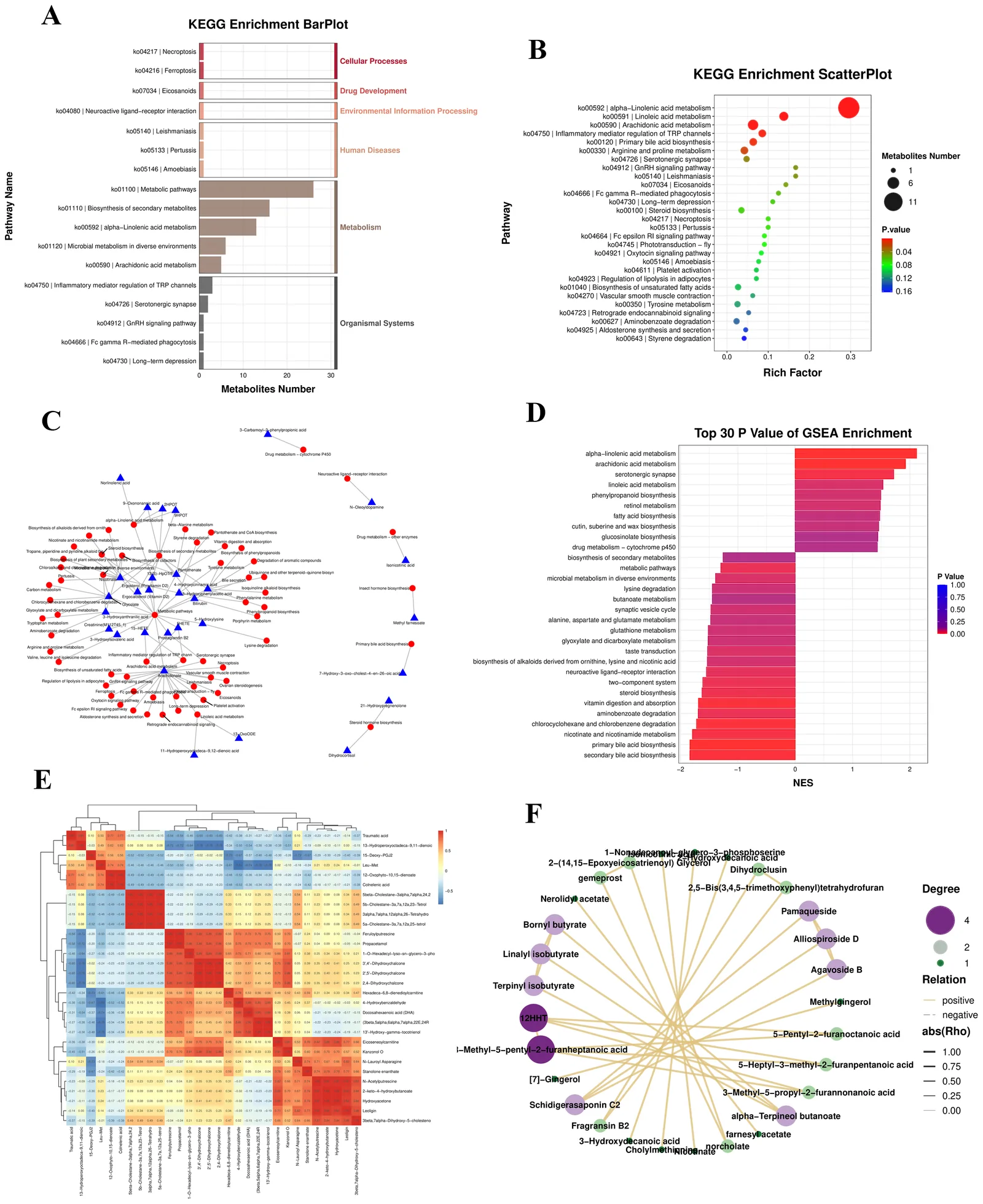
Functional analysis of differential metabolites in Malaysian pangolins infected with CPV and healthy individuals. **(A, B)** Metabolite enrichment pathways by KEGG analysis. **(C)** KEGG pathway and metabolite network diagram. **(D)** The top 30 metabolites with the lowest P value after GSEA analysis of KEGG pathways. **(E, F)** Heat map and network map of correlation analysis among the top 30 significant differential metabolites with the lowest P value.

To investigate the biological processes involved in the differentially expressed metabolites induced by CPV infection, this study performed KEGG pathway enrichment analysis and gene set enrichment analysis (GSEA). KEGG enrichment analysis (**Figures 2A, B**) revealed that differentially expressed metabolites were significantly enriched in pathways such as α-linolenic acid metabolism, arachidonic acid metabolism, linoleic acid metabolism, ferroptosis, necrotic apoptosis, neuroactive ligand-receptor interactions, and serotonergic synapses. The metabolite-pathway association network (**Figure 2C**) showed that differentially expressed metabolites such as 15-HETE, 13-Oxo-DE, prostaglandin B2, methylfarnesyl ester, and 7-hydroxy-3-oxocholest-4-ene-26-acid were closely associated with the above pathways. GSEA analysis (**Figure 2D**) revealed that α-linolenate metabolism, arachidonic acid metabolism, serotonergic synapses, linoleic acid metabolism, retinol metabolism, and fatty acid biosynthesis were significantly enriched in the CPV-infected group (P < 0.05). Correlation analysis of differentially expressed metabolites (**Figures 2E, F**) revealed that metabolites such as 12HHT, furan fatty acid derivatives, and acetate esters formed multiple highly correlated clustering modules, with fatty acid-related metabolites exhibiting predominantly positive correlations.

### 3.3 Differential analysis of metabolites between Chinese pangolins infected with CPV and healthy individuals

**Figure 3:**
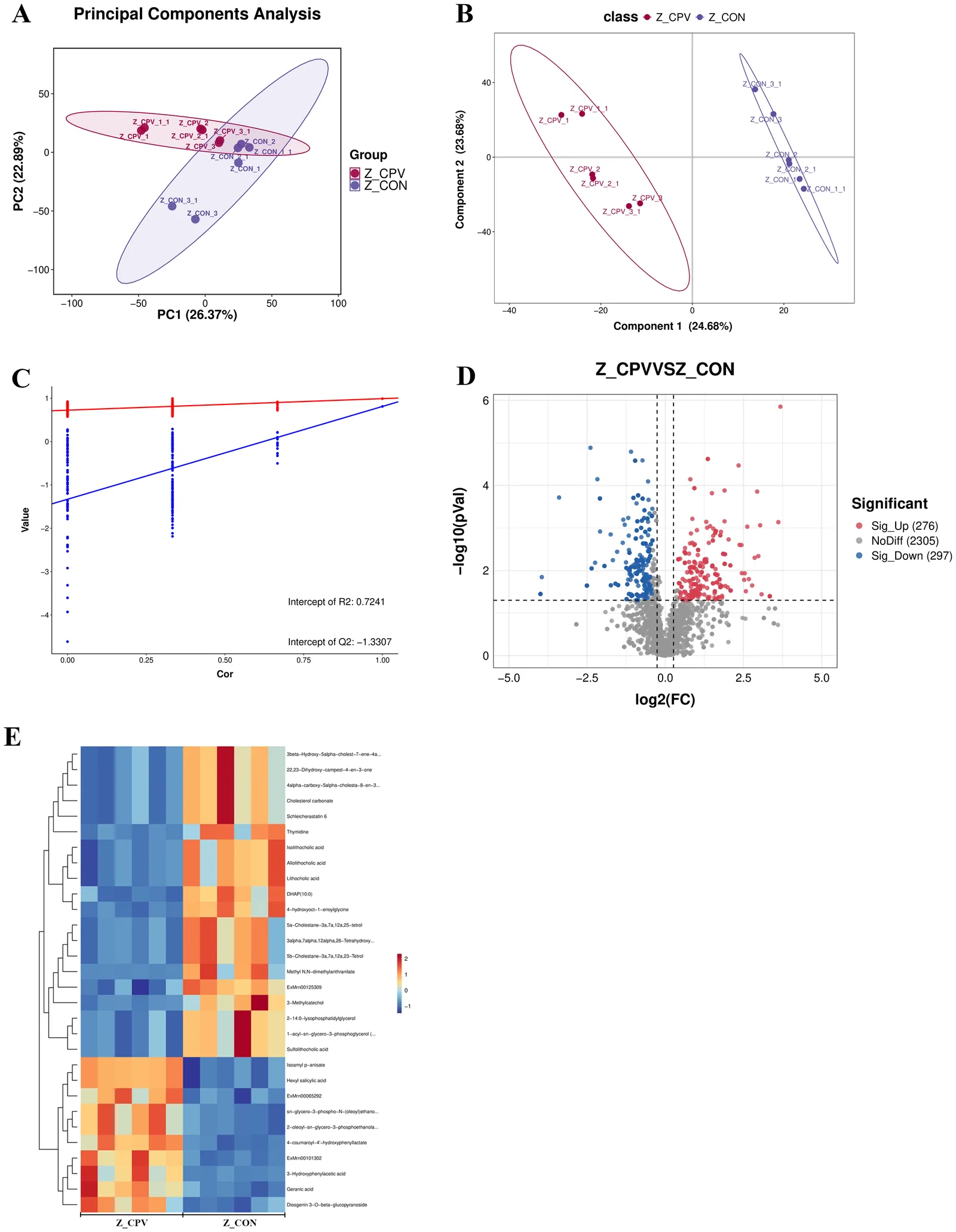
Differential analysis of metabolites between Chinese pangolins infected with CPV and healthy individuals. **(A)** PCA analysis. **(B, C)** PLSDA analysis and permutation test plot. **(D)** Volcano map of differential metabolites. **(E)** Heat map of differential metabolites.

To investigate the effects of CPV infection on fecal metabolites in the Chinese pangolin, this study also performed non-targeted metabolomics analysis on samples from the CPV-infected group (Z_CPV) and the healthy control group (Z_CON). PCA results showed that PC1 accounted for 26.37% of the variance and PC2 for 22.89%; the two groups of samples exhibited a clear separation trend in the score plot (**Figure 3A**). The PLSDA cross-validation results indicated that the model was not overfitted and the results were reliable. Meanwhile, the PLSDA score plot results showed significant differences in metabolite expression patterns between the CPV-infected group (Z_CPV) and the control group (Z_CON) (**Figures 3B, C**). Taking FC>1.2 and P<0.05 as the screening criteria, a total of 573 significantly differentially expressed metabolites were identified, of which 276 were upregulated and 297 were downregulated in the CPV-infected group (**Figure 3D**). The heat map of differentially expressed metabolites demonstrated distinct clustering between the Z_CPV and Z_CON metabolic profiles (**Figure 3E**).

### 3.4 Functional analysis of differential metabolites in Chinese pangolins infected with CPV and healthy individuals

**Figure 4:**
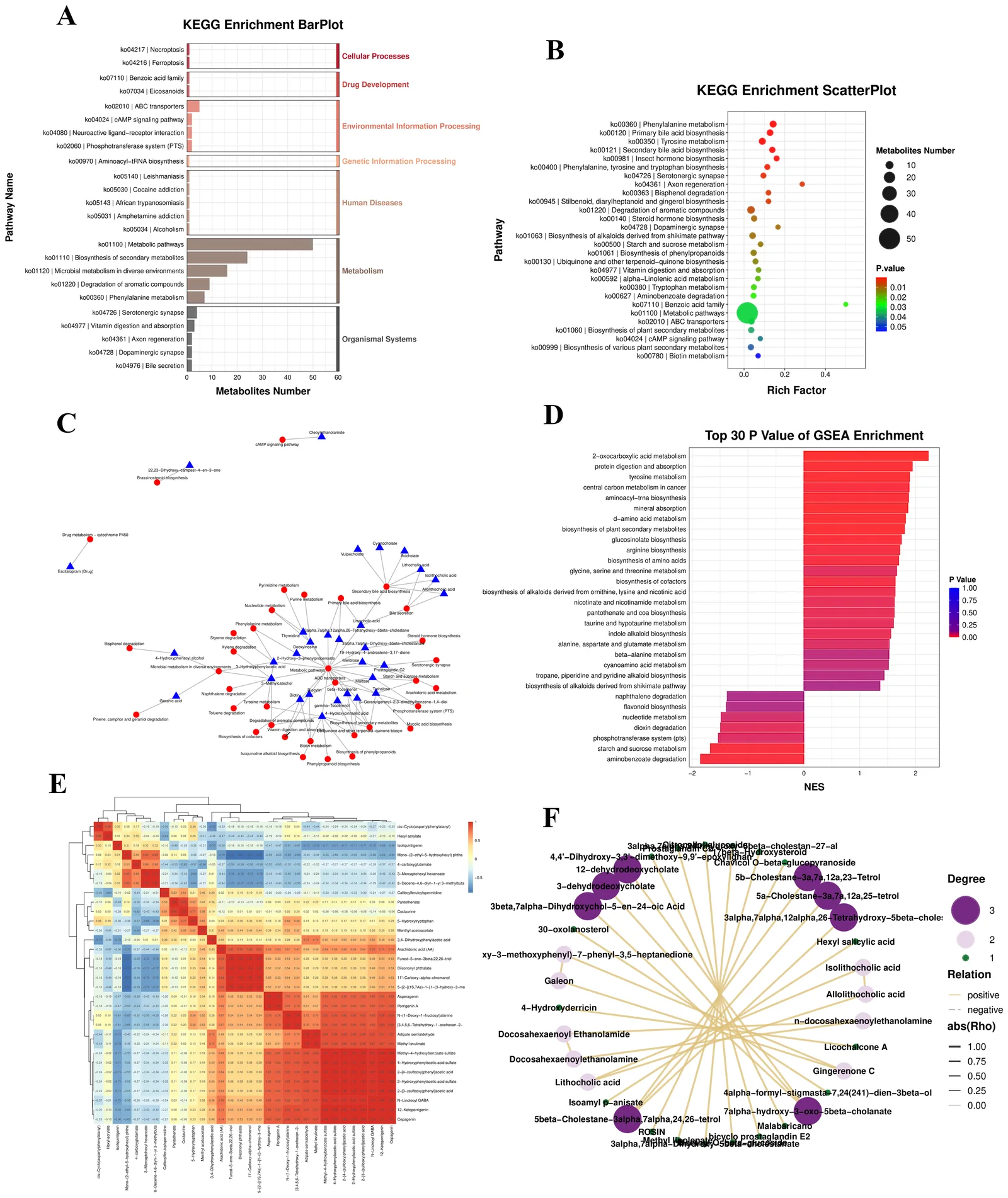
Functional analysis of differential metabolites in Chinese pangolins infected with CPV and healthy individuals. **(A, B)** Metabolite enrichment pathways by KEGG analysis. **(C)** KEGG pathway and metabolite network diagram. **(D)** The top 30 metabolites with the lowest P value after GSEA analysis of KEGG pathways. **(E, F)** Heat map and network map of correlation analysis among the top 30 significant differential metabolites with the lowest P value.

KEGG enrichment analysis (**Figures 4A, B**) revealed that metabolites differentially expressed in Chinese pangolins following CPV infection were significantly enriched in multiple metabolic pathways, including necrotic apoptosis, ferroptosis, arachidonic acid metabolism, neuroactive ligand-receptor interactions, and cAMP signaling. GSEA analysis (**Figure 4D**) further confirmed that pathways such as 2-oxocarboxylic acid metabolism, protein digestion and absorption, tyrosine metabolism, and aminoacyl-tRNA biosynthesis were significantly enriched in the CPV-infected group (Z_CPV) (P < 0.05). The metabolite-pathway association network (**Figure 4C**) shows that differentially expressed metabolites such as oleoylthionalamide, vulpecholate, and escitalopram are closely associated with pathways including the cAMP signaling pathway, pyrimidine metabolism, and brassinosteroid biosynthesis. In the correlation analysis of differentially expressed metabolites (**Figures 4E, F**), major metabolite nodes included 3α,7α-dihydroxy-5β-cholestane, deoxycholic acid derivatives, tetrahydroxycholanoic acid, and various cholesterol metabolic intermediates (such as 3-oxo-4-ene compounds). These bile acid-related metabolites primarily exhibit positive correlations, forming highly interconnected clustering modules.

### 3.5 Differential analysis of metabolites in Chinese pangolins and Malaysian pangolins after CPV infection

**Figure 5:**
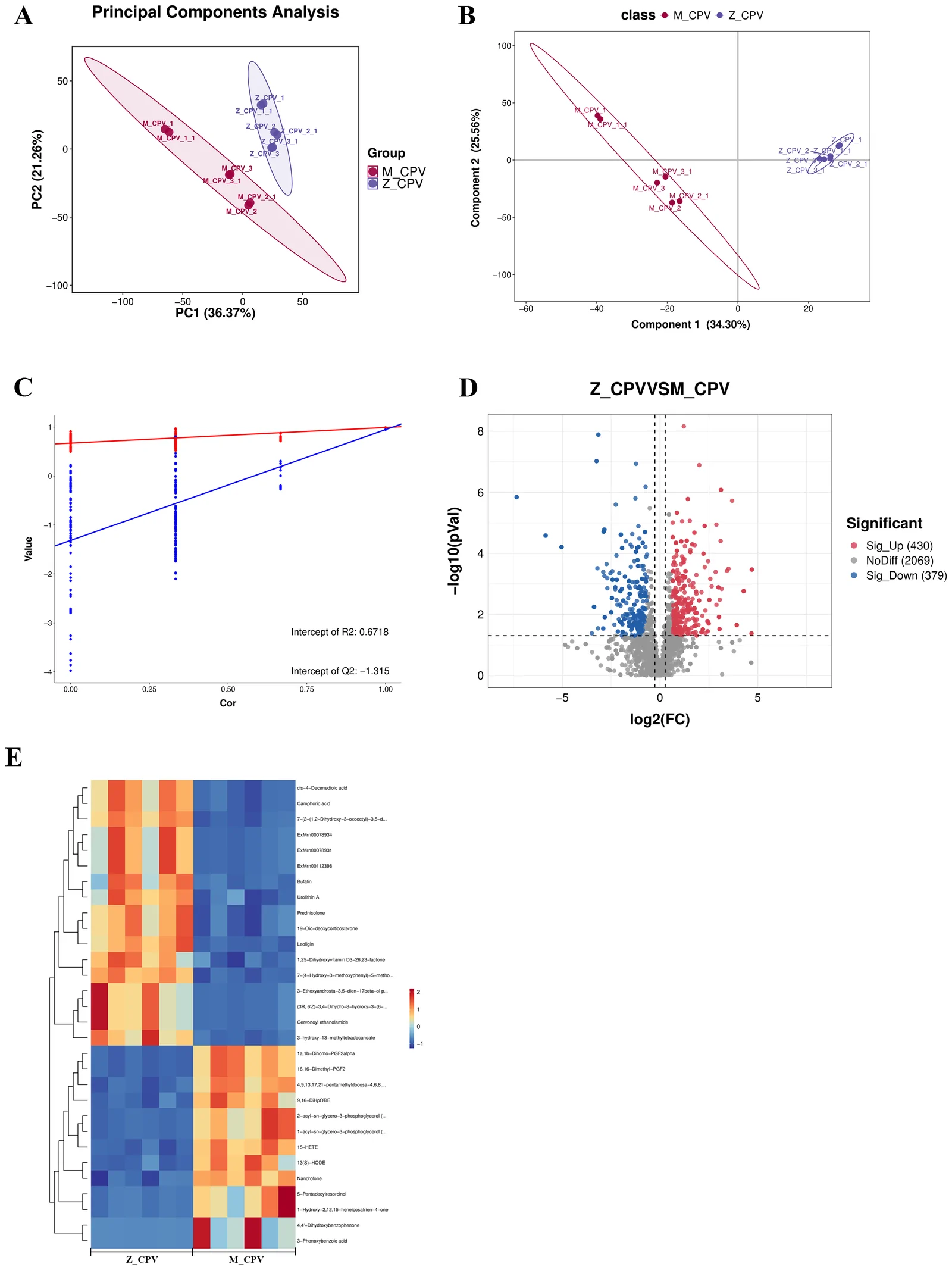
Differential analysis of metabolites in Chinese pangolins and Malaysian pangolins after CPV infection. **(A)** PCA analysis. **(B, C)** PLSDA analysis and permutation test plot. **(D)** Volcano map of differential metabolites. **(E)** Heat map of differential metabolites.

To compare the differences in metabolic responses between the two pangolin species following CPV infection, this study conducted a direct comparative analysis of fecal metabolites from the Malaysian pangolin infection group (M_CPV) and the Chinese pangolin infection group (Z_CPV). PCA results showed that the contribution rate of PC1 was 36.37%, and that of PC2 was 21.28%. The two groups of samples showed an obvious separation trend in the score chart (**Figure 5A**). The PLSDA permutation test plot showed that the R2 intercept was 0.6718 and the Q2 intercept was -1.315, indicating that the model was not overfitted (**Figure 5C**). Meanwhile, the PLSDA score plot results showed that samples from the Z_CPV group and the M_CPV group clustered in different regions, with clear boundaries between the groups, indicating significant differences in metabolite expression patterns between the two groups (**Figure 5B**). Using a screening criterion of FC > 1.2 and P < 0.05, a total of 809 significantly differentially expressed metabolites were identified, of which 430 were upregulated and 379 were downregulated in the Z_CPV group relative to the M_CPV group (**Figure 5D**). The heat map of differentially expressed metabolites (**Figure 5E**) shows distinct clustering of the two groups in their metabolic profiles. The M_CPV group had high expression of fatty acid derivatives (such as 15-HETE, prostaglandins), plant-derived compounds (such as Agavoside B) and terpenoids. Z_CPV group was relatively enriched in bile acids (such as chenodeoxycholic acid derivatives, deoxycholic acid), amino acid metabolites (such as 5-hydroxylysine) and aromatic compounds (such as 3-hydroxyphenylacetic acid).

### 3.6 Functional analysis of differential metabolites in Chinese pangolins and Malaysian pangolins after CPV infection

**Figure 6:**
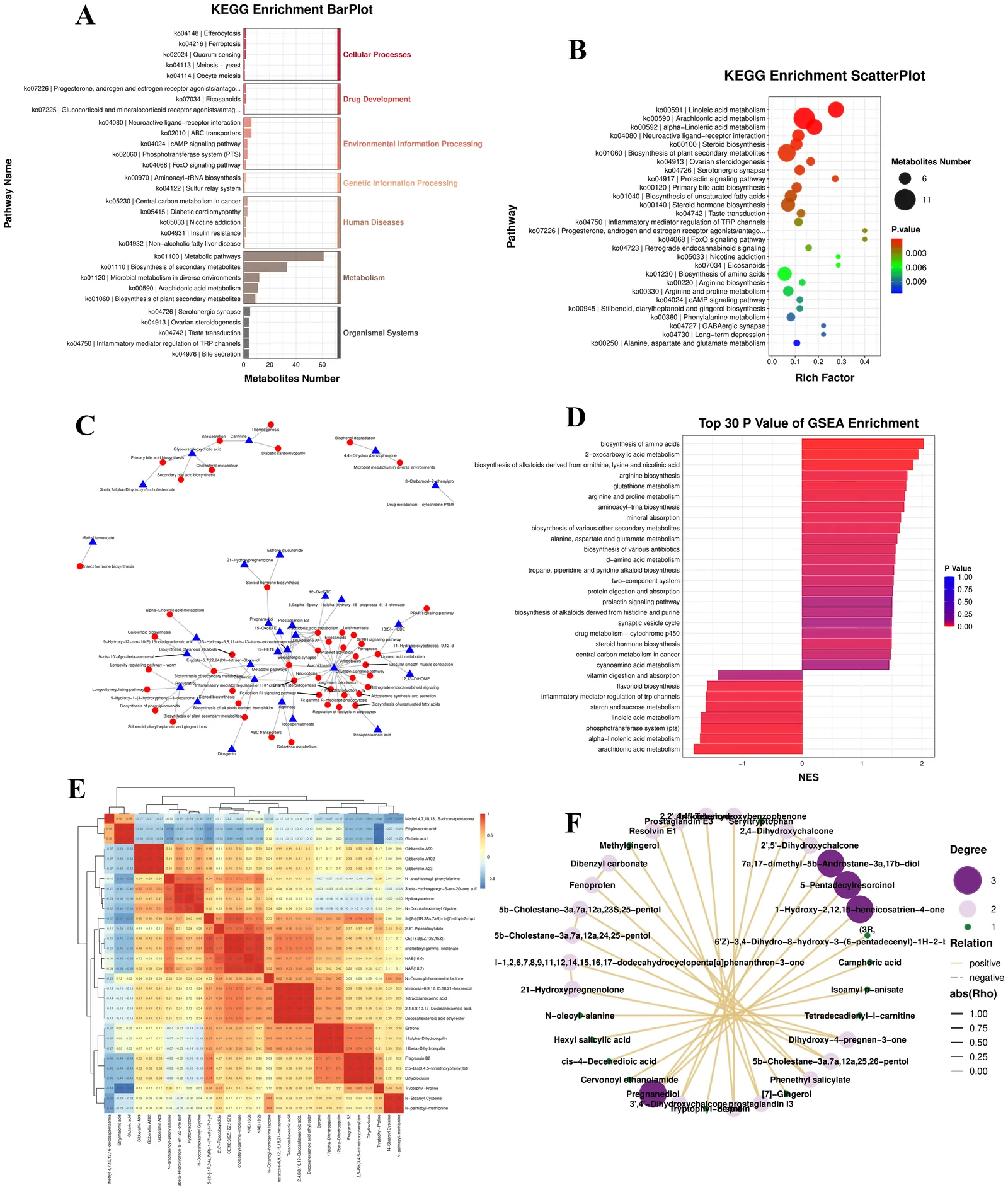
Functional analysis of differential metabolites in Chinese pangolins and Malaysian pangolins after CPV infection. **(A, B)** Metabolite enrichment pathways by KEGG analysis. **(C)** KEGG pathway and metabolite network diagram. **(D)** The top 30 metabolites with the lowest P value after GSEA analysis of KEGG pathways. **(E, F)** Heat map and network map of correlation analysis among the top 30 significant differential metabolites with the lowest P value.

KEGG enrichment analysis revealed that differential metabolites between the Z_CPV and M_CPV groups were significantly enriched in linoleic acid metabolism, arachidonic acid metabolism, α-linolenic acid metabolism, and neuroactive ligand receptor interaction (**Figures 6A, B**). GSEA analysis further showed that amino acid biosynthesis, 2-oxocarboxylic acid metabolism, arginine biosynthesis, and glutathione metabolism were significantly differentially enriched between the two groups (**Figure 6D**). The metabolite-pathway association network diagram showed that multiple lipid metabolism-related pathways, such as arachidonic acid metabolism, α-linolenic acid metabolism, and linoleic acid metabolism, were closely associated with key metabolites including 15-HETE, prostaglandin B2, arachidonic acid, and glycocholic acid (**Figure 6C**). In the correlation analysis of differentially expressed metabolites, steroidal hormones (such as androgens and pregnane derivatives) and bile acid metabolites served as core nodes, forming a highly interconnected network structure (**Figures 6E, F**).

## 4 Discussion

In this study, we systematically analyzed the differences in fecal metabolites between Malaysian pangolins and Chinese pangolins before and after CPV infection using untargeted metabolomics techniques. The results indicate that CPV infection significantly altered the fecal metabolomic profiles of both pangolin species. Differentially expressed metabolites were primarily enriched in lipid metabolism, amino acid metabolism, energy metabolism, and inflammation-related pathways, and there were marked interspecies differences in the metabolic response characteristics between the two pangolin species.

In Malaysian pangolins, the metabolic profiles of the CPV-infected group and the healthy control group were significantly separated, and the differential metabolites were mainly enriched in polyunsaturated fatty acid metabolic pathways, including α-linolenic acid, arachidonic acid, and linoleic acid metabolism. Arachidonic acid metabolism is a major source of inflammatory mediators, and its abnormal activation is usually closely related to the inflammatory response induced by viral infection (Chandrasekharan et al., 2016). In this study, compared to the M_CON group, arachidonic acid derivatives such as 15-HETE and prostaglandin B2 were significantly upregulated in the M_CPV group, suggesting that CPV infection may induce intestinal inflammation by activating the arachidonic acid cascade. This finding is consistent with studies showing elevated prostaglandin levels and impaired barrier function in the intestinal mucosa of CPV-infected dogs (Atkinson et al., 2022; Panda et al., 2009). Furthermore, the enrichment of ferroptosis and necroptosis pathways suggests that CPV infection may exacerbate intestinal epithelial damage through non-apoptotic cell death pathways. Ferroptosis is a cell death mode driven by iron-dependent lipid peroxidation (Gao et al., 2025), while necroptosis is mediated by receptor-interacting protein kinases 1 and 3 (RIPK1/RIPK3) and can trigger intense inflammation (Peng, 2024). This is consistent with the pathological features of severe hemorrhagic enteritis frequently observed in dogs infected with CPV, suggesting that lipid peroxidation and programmed necrosis may be key mechanisms underlying CPV-induced intestinal epithelial damage. Correlation analysis revealed that metabolites such as 12HHT, furan fatty acid derivatives, and acetate esters form multiple highly correlated clustering modules, with fatty acid-related metabolites exhibiting predominantly positive correlations, further demonstrating that CPV infection may synergistically regulate multiple lipid metabolic pathways.

Different from the Malaysian pangolin, the Chinese pangolin was significantly enriched in not only lipid metabolism pathways, but also amino acid metabolism-related pathways after CPV infection. KEGG analysis showed that differentially expressed metabolites were significantly enriched in pathways such as phenylalanine metabolism, tyrosine metabolism, and arginine and proline metabolism. GSEA analysis further confirmed that 2-oxocarboxylic acid metabolism, tyrosine metabolism, and aminoacyl-tRNA biosynthesis were significantly enriched in Z_CPV. Aromatic amino acids such as phenylalanine and tyrosine serve as precursors for the synthesis of neurotransmitters (such as dopamine and norepinephrine), and abnormalities in their metabolism may be associated with neuroendocrine disturbances caused by viral infection (Anand et al., 2024; Gietl et al., 2023). Basoglu’s team (Basoglu et al., 2025) similarly found that dogs infected with CPV exhibited changes in the levels of various amino acids, including decreased levels of glutamate and glycine and increased levels of isoleucine, isovaleric acid, and creatine. CPV infection causes damage to the intestinal mucosal barrier, villous atrophy, and crypt destruction (Macartney et al., 1984), severely impairing amino acid absorption. The accompanying protein-losing enteropathy and decreased hepatic synthetic function may further exacerbate amino acid metabolic disorders (Paul et al., 2023). In addition, correlation analysis showed that bile acid-related metabolites form highly interconnected clustering modules in Chinese pangolins, suggesting that CPV infection may disrupt bile acid metabolism in these animals. Bile acids not only assist lipid digestion and absorption, but also act as signaling molecules to regulate inflammatory responses and energy metabolism by activating the farnesoid X receptor (FXR) and the G protein-coupled bile acid receptor 5 (TGR5) (Jia et al., 2025). Related studies have shown that viral infection affects the expression of the intestinal FXR signaling pathway, leading to increased bile acid synthesis and impaired intestinal barrier function (Liu et al., 2023; Romain et al., 2025). The significant enrichment of bile acid metabolites in the infected Chinese pangolin group may reflect this species’ unique response mechanism to CPV infection.

A direct comparison of the metabolic profiles of the two pangolin CPV infection groups, followed by functional analysis, revealed that the differentially expressed metabolites between the two groups were enriched in pathways such as linoleic acid metabolism, arachidonic acid metabolism, α-linolenic acid metabolism, and neuroactive ligand-receptor interactions. Fatty acid derivatives and terpenoids were relatively highly expressed in the Malaysian pangolin infected group, while bile acids and amino acid metabolites were relatively enriched in the Chinese pangolin infected group. Correlation network analysis revealed that steroid hormones and bile acid metabolites formed a highly interconnected core module, suggesting that the two pangolin species initiated distinct metabolic reprogramming programs following CPV infection. This interspecific difference may result from a variety of factors. On the one hand, the Chinese pangolin and the Malaysian pangolin diverged approximately millions of years ago, and differences at the genomic level may influence their overall metabolic response to viral infection (Choo et al., 2016). On the other hand, the pangolins’ specialized diet results in a highly specialized gut microbiota (Brunetti et al., 2023), and differences in microbial community structure between species may directly affect the composition of fecal metabolites. Furthermore, the Malaysian pangolin is primarily distributed in lowland tropical rainforests of Southeast Asia, whereas the Chinese pangolin has a broader range, encompassing subtropical and temperate regions. This long-term ecological niche divergence may have led to adaptive differences between the two pangolin species in terms of energy metabolism and stress adaptation strategies.

## 5 Conclusion

CPV infection significantly affected the fecal metabolic profiles of the two pangolins, and the differential metabolites were mainly enriched in lipid metabolism (especially polyunsaturated fatty acid metabolism), amino acid metabolism, and inflammation-related pathways (ferroptosis and necroptosis). The Malaysian pangolin is characterized by lipid metabolism disorder and inflammatory response after infection, while the Chinese pangolin shows more prominent reprogramming of amino acid metabolism and bile acid metabolism disorder. The interspecies differences in metabolic responses between the two species may stem from variations in genetics and gut microbiota composition. This study provides potential fecal metabolic biomarkers for the early identification of CPV infection in pangolins, offering a new theoretical basis for disease monitoring and intervention in pangolin conservation.

## Data Availability

The data used to support the findings of this study are available from the corresponding author upon reasonable request. The raw metabolomics data have been deposited in the MetaboLights database under the accession number MTBLS15426. The study is accessible at the following URL: https://www.ebi.ac.uk/metabolights/MTBLS15426.
